# Sociodemographic Patterns of Exclusive and Dual Use of ENDS and Menthol/Non-Menthol Cigarettes among US Youth (Ages 15–17) Using Two Nationally Representative Surveys (2013–2017)

**DOI:** 10.3390/ijerph18157781

**Published:** 2021-07-22

**Authors:** Akash Patel, Jana L. Hirschtick, Steven Cook, Bukola Usidame, Ritesh Mistry, David T. Levy, Rafael Meza, Nancy L. Fleischer

**Affiliations:** 1 Center for Social Epidemiology and Population Health, Department of Epidemiology, School of Public Health, University of Michigan, Ann Arbor, MI 48109, USA; janahirs@umich.edu (J.L.H.); cookstev@umich.edu (S.C.); busidame@umich.edu (B.U.); nancyfl@umich.edu (N.L.F.); 2 Department of Health Behavior and Health Education, School of Public Health, University of Michigan, Ann Arbor, MI 48109, USA; riteshm@umich.edu; 3 Lombardi Comprehensive Cancer Center, Georgetown University, Washington, DC 20007, USA; dl777@georgetown.edu; 4 Department of Epidemiology, School of Public Health, University of Michigan, Ann Arbor, MI 48109, USA; rmeza@umich.edu

**Keywords:** tobacco, cigarettes, electronic nicotine delivery systems (ENDS), population, adolescents, youth, epidemiology

## Abstract

The use of electronic nicotine delivery systems (ENDS) among youth in the United States has increased rapidly in the past decade. Simultaneously, while youth cigarette smoking has declined considerably, youth are still more likely to use menthol cigarettes than any other age group. We used nationally representative data on 15–17-year-olds from the Population Assessment of Tobacco and Health (PATH) Study and the National Youth Tobacco Survey (NYTS) (2013–2017) to better understand current cigarette (by menthol flavoring) and ENDS use in the US. We calculated weighted population prevalence estimates across years for multiple patterns of current cigarette and ENDS use (i.e., exclusive menthol cigarette, exclusive non-menthol cigarette, exclusive ENDS, dual ENDS and menthol cigarette, and dual ENDS and non-menthol cigarette) by sex, race/ethnicity, parental education level, household income, and homeownership. Overall, both exclusive menthol and non-menthol cigarette use declined from 2013–2017. Exclusive ENDS use increased, particularly among youth who were non-Hispanic White or had a higher socioeconomic status (measured by parental education, household income, and homeownership). Dual use of ENDS with either menthol or non-menthol cigarettes did not change significantly. Monitoring changes in these sociodemographic patterns will help inform future youth tobacco prevention strategies.

## 1. Introduction

Over the past 20 years, there has been a continued reduction in cigarette smoking among middle and high school-aged youth (9–19 years) in the United States [1,2,3]. A gradual decline in youth non-menthol cigarette use occurred from 2004 to 2010, but the prevalence of menthol cigarette use remained relatively stable during the same period [4,5,6,7]. In 2009, the US Food and Drug Administration (FDA) gained the authority to regulate the manufacturing, distribution, and marketing of tobacco products when the Family Smoking Prevention and Tobacco Control Act was signed into law [8]. Among other provisions, the law prohibited the sale of flavored cigarettes, excluding menthol and tobacco, restricted tobacco advertising that was considered attractive to youth (e.g., banning free giveaways of sample cigarettes), and required the FDA to at least regulate cigarette tobacco, roll-your-own tobacco, cigarettes, and smokeless tobacco [8]. In more recent years, the prevalence of non-cigarette tobacco and nicotine product use, particularly electronic nicotine delivery systems (ENDS), has increased dramatically among youth [2,9,10,11]. The FDA issued a final rule in 2016 to deem all products that met the definition of a tobacco product, including ENDS, cigars, pipes, and hookah, subject to its regulatory authority [12]. Since then, it has started enforcing the raised federal minimum age of sale for all tobacco products from 18 to 21 years in December 2019 [13], banned all flavored ENDS cartridges with the exception of menthol and tobacco flavors in January 2020 [14], and proposed banning menthol cigarettes and all flavored cigars in April 2021 [15]. Better understanding and closely monitoring changes to youth tobacco use patterns is necessary with significant changes occurring in the tobacco marketplace and with US health policy. Literature examining patterns of exclusive and dual use of ENDS and menthol/non-menthol cigarettes among youth, over time in particular, is limited.

Menthol cigarettes have been disproportionately promoted among populations at high risk [4,16], including youth and young adults, which may be a key reason why they are more prevalent among younger age groups [6,7]. As ENDS use started to rise in 2011–2013 and overall cigarette smoking began to decrease rapidly among youth [3,17], menthol cigarette use also began to decline at a rate comparable to non-menthol cigarette use in prior years [6,18]. From 2011 to 2016, the prevalence of menthol cigarette use significantly decreased from 6.2 to 2.6%, whereas the prevalence of non-menthol cigarette use decreased from 4.1 to 2.5% over the same period among all US middle and high school students; from 2016 to 2018, prevalence of menthol cigarette use (2.6 to 2.5%) and non-menthol cigarette use (2.5 to 2.5%) did not change [18]. Youth menthol cigarette use is concerning because menthol cigarette smokers appear to be more likely than non-menthol cigarette smokers to smoke more cigarettes [19], intend to continue smoking [19], progress to regular smoking [20,21], and become nicotine dependent [20,22,23,24]. In addition to increasing the reinforcing effects of nicotine [24], menthol-flavored cigarettes appeal to newer, younger smokers who find them less harsh to smoke than non-menthol cigarettes [22,23,25,26].

ENDS products have been marketed as less harmful alternatives to combustible tobacco products [27] and are available in a variety of flavors that are appealing to adult smokers trying to switch, as well as youth interested in trying ENDS [28,29,30,31,32]. In recent years, there has been an increase in past 30-day ENDS use among youth; the prevalence of current ENDS use among US high school students increased from 1.5% in 2011 to 27.5% in 2019, then decreased to 19.6% in 2020 [33,34,35]. There are also important variations across sociodemographic subgroups; for example, older, Hispanic, and White youth are more likely to use ENDS than younger and Black youth [36]. It is unclear how the rapid increase in ENDS use among youth will impact long-term tobacco use patterns in the future. There is a contentious debate over the relationship between ENDS use and cigarette use among youth; some researchers contend that ENDS use may act as a “gateway” to future cigarette smoking [37,38], whereas others maintain that common liability factors such as genes and risk-taking behaviors predispose some individuals to use ENDS and cigarettes, regardless of order [39,40]. Several longitudinal studies have found a positive association between ENDS use and subsequent initiation of cigarette smoking among youth [41,42,43,44,45,46,47,48,49,50,51], but systematic reviews note that short follow-up periods [47], inadequate adjustment for confounding variables (e.g., common liability factors) [43,49,50], high rates of attrition [43,50], and publication bias [49,50] limit the conclusions we can draw from these findings. Moreover, while ENDS use among youth has increased dramatically, the rate of youth cigarette smoking has reached a historic low [3,52]. In the absence of more robust longitudinal studies on the transitional states between youth ENDS and cigarette use, studies assessing trends in the prevalence of exclusive and dual use of ENDS and menthol/non-menthol cigarettes can provide important data.

Current literature tends to categorize menthol and non-menthol cigarette users together when examining patterns of dual and polytobacco use. However, each combination of ENDS and menthol/non-menthol cigarette use may have distinct sociodemographic profiles that have not been studied. The health impact of using ENDS with menthol cigarettes versus non-menthol cigarettes is not well known, but monitoring current changes in cigarette and ENDS use patterns can help accurately identify groups most likely to be impacted, inform future tobacco prevention strategies targeting youth, and make better predictions on the impact of new policy implementation. Examining trends by menthol and non-menthol cigarette use is also important with the recent proposal by the FDA to ban menthol cigarettes [15]. Using two nationally representative surveys with different modes of administration from 2013 to 2017, we examined the overall trends of exclusive menthol cigarette use, exclusive non-menthol cigarette use, exclusive ENDS use, dual use of ENDS and menthol cigarettes, and dual-use of ENDS and non-menthol cigarettes among adolescents aged 15–17. Additionally, we examined differences in use by sociodemographic groups (i.e., sex, race/ethnicity, parental educational attainment, annual household income, and homeownership). To the best of our knowledge, no prior studies have examined trends in exclusive and dual use of ENDS and menthol/non-menthol cigarettes among 15–17-year-olds.

## 2. Methods

### 2.1. Data

We used data from four waves (2013/14–2016/17) of the Population Assessment of Tobacco and Health (PATH) Study and five years (2013–2017) of the National Youth Tobacco Survey (NYTS) to estimate the population prevalence of exclusive and dual use of ENDS and menthol/non-menthol cigarettes among 15–17-year-olds. We chose to restrict our analysis to this age group, which corresponds to high school-aged adolescents because it is a critical time in which most youth initiate tobacco use and some escalate from experimental use to established use [53].

The PATH Study is a collaboration between the National Institutes of Health, through the National Institute on Drug Abuse (NIDA), and the FDA’s Center for Tobacco Products [54,55]. It is an ongoing, nationally representative longitudinal cohort study that employs a four-stage stratified area probability sampling design to recruit participants from the civilian, non-institutionalized US population ages 12 and older [54,55]. Youth (ages 12–17) and adults (ages 18+) answer questions about tobacco product use in English or Spanish using audio computer-assisted self-interviewing (ACASI). ACASI allows participants to hear questions using headphones, simultaneously read questions on a screen, and directly enter their responses using a touch screen or keyboard. Except for emancipated youth, both consent from a parent or legal guardian and youth assent were required to conduct youth interviews [54,55]. The parent or legal guardian who provided consent was also asked to complete a brief parent interview in regards to their participating youth and household demographics (e.g., their education level, annual household income, and homeownership) using computer-assisted personal interviewing (CAPI) [55]. Unlike ACASI, CAPI is a data collection method where an interviewer conducts face-to-face interviews with participants and directly enters responses into a laptop or tablet.

This study reports weighted cross-sectional prevalence estimates from PATH 2013/14 (Wave 1; 12 September 2013–14 December 2014; *n* = 6596), PATH 2014/15 (Wave 2; 23 October 2014–30 October 2015; *n* = 5858), PATH 2015/16 (Wave 3; 19 October 2015–23 October 2016; *n* = 5782), and PATH 2016/17 (Wave 4; 1 December 2016–3 January 2018; *n* = 7414). PATH 2013/14 and PATH 2016/17 are nationally representative, whereas PATH 2014/15 and PATH 2015/16 are pseudo-nationally representative. In PATH 2016/17, a probability sample of adults, youth, and shadow youth selected from the civilian, non-institutionalized US population at the time of the wave (i.e., a replenishment sample) was added to the cohort of continuing participants from PATH 2013/14 to ensure PATH 2016/17 was nationally representative [55].

NYTS is a cross-sectional, nationally representative survey of middle and high school students (grades 6 through 12) selected using a stratified, three-stage cluster design of public and private schools in all 50 US states and the District of Colombia [56,57,58,59,60]. It is a self-administered survey completed in school where participation is voluntary at both the school and student levels. Parental consent and student assent were required to participate, but unlike PATH, parents or legal guardians were not asked to complete any survey regarding their participating youth and household demographics. This study reports weighted cross-sectional prevalence estimates from 2013 (12 February 2013–21 June 2013; *n* = 7363), 2014 (10 February 2014–13 June 2014; *n* = 8460), 2015 (9 February 2015–19 June 2015; *n* = 7088), 2016 (8 February 2016–7 June 2016; *n* = 8095), and 2017 (13 February 2017–14 June 2017; *n* = 7582).

Since we used de-identified publicly available PATH and NYTS datasets, this study was classified as not regulated human subjects research by the University of Michigan Institution Review Board.

### 2.2. Measures

#### 2.2.1. Tobacco Products

We defined current cigarette use as having smoked cigarettes at least one day in the past 30 days, using the following questions: “In the past 30 days, on how many days did you smoke cigarettes?” (PATH), or “During the past 30 days, on how many days did you smoke cigarettes?” (NYTS). Among current cigarette smokers, we defined menthol cigarette use as affirmative responses to: “In the past 30 days, were any of the cigarettes you smoked flavored to taste like menthol or mint?” (PATH) or “During the past 30 days, were the cigarettes that you usually smoked menthol?” (NYTS).

We defined current ENDS use as having used ENDS at least one day in the past 30 days, based on the questions: “In the past 30 days, on how many days did you use an electronic product?” (PATH) or “During the past 30 days, on how many days did you use e-cigarettes?” (NYTS).

We then classified participants into six mutually exclusive use patterns—never/non-current use, exclusive menthol cigarette use, exclusive non-menthol cigarette use, exclusive ENDS use, dual use of ENDS and menthol cigarettes, and dual-use of ENDS and non-menthol cigarettes. We grouped participants who were missing information about menthol flavoring (*n* = 0–43 for PATH and *n* = 531–606 for NYTS across waves) with non-menthol cigarette smokers to preserve our overall estimates of cigarette use prevalence. Lastly, if we could not ascertain participants’ current cigarette and/or ENDS use status, we categorized them as missing (0.5–0.8% PATH; 0.8–2.1% NYTS).

#### 2.2.2. Sociodemographic Characteristics

We stratified analyses by sex (male, female) and race/ethnicity (Non-Hispanic (NH) White, NH Black, Hispanic, NH Other) in both NYTS and PATH. We also stratified estimates by parental education level (high school/GED or less, some college, college or more), annual household income (<$50,000, ≥$50,000—not available for PATH 2013/14), and homeownership (owned, not owned—not available for PATH 2013/14) in PATH only, as these indicators of socioeconomic status were not available in NYTS.

### 2.3. Statistical Analysis

We conducted all analyses using Stata software, version 16 (StataCorp). For both PATH and NYTS, we accounted for the complex sample design and calculated weighted population prevalence of current exclusive or dual use of ENDS and menthol or non-menthol cigarettes among 15–17-year-olds overall and by sex, race/ethnicity, parental education, household income, and homeownership. Given the large number of potential comparisons in the prevalence of the six category use pattern variable across 13 sociodemographic strata and two surveys, we evaluated significant differences in prevalence of tobacco use patterns between sociodemographic subgroups and over time, using estimates from 2013 and 2017, by examining confidence interval overlap. We used the balanced repeated replication method with Fay’s adjustment of 0.3 for PATH [61] and Taylor series linearization for NYTS to estimate variances and confidence intervals [62].

## 3. Results

### 3.1. Sample Demographics

Table 1 summarizes the sample characteristics and overall distribution of exclusive and dual use of cigarettes and ENDS from 2013 to 2017 in PATH and NYTS. Both surveys had similar sex and race/ethnicity distributions. The sample size of 15–17-year-olds in PATH 2013/14 was 6596 (51.2% male, 55.5% NH White and 21.9% Hispanic); PATH 2016/17 included a replenishment sample that increased the sample size to 7414 (51.0% male, 53.5% NH White and 23.2% Hispanic, 56.4% with household income ≥$50,000). In NYTS, the sample of 15–17-year-olds was 7363 in 2013 (50.7% male, 53.6% NH White, and 21.3% Hispanic), and 7582 in 2017 (50.8% male, 53.4% NH White, and 23.2% Hispanic).

### 3.2. Prevalence Estimates of ENDS and Menthol/Non-Menthol Cigarette Use

Exclusive ENDS use was the only tobacco use pattern examined that significantly increased from 2013 to 2017 across both surveys—from 2.7 to 5.0% in PATH and 1.5 to 7.3% in NYTS (Table 1, Tables S1 and S2, Figure S1). Conversely, exclusive menthol cigarette use decreased in both surveys (PATH: 3.1 to 1.6%; NYTS: 3.7 to 1.5%) and exclusive non-menthol cigarette use decreased in NYTS (4.9 to 1.9%), but not significantly in PATH (2.1 to 1.8%). The prevalence of both dual-use patterns did not change significantly in either survey. However, by 2017, in NYTS, the prevalence of each dual-use pattern (ENDS and menthol cigarette: 1.6% and ENDS and non-menthol cigarette: 2.3%) was comparable to exclusive cigarette use prevalence (exclusive menthol cigarettes: 1.5% and exclusive non-menthol cigarettes: 1.9%).

### 3.3. Prevalence Estimates by Sex

Trends in ENDS and menthol/non-menthol cigarette use from 2013–2017 were generally similar by sex (Tables S3 and S4, Figure S2). For both males and females, exclusive ENDS use increased while exclusive menthol and non-menthol cigarette use generally decreased. One notable difference was in dual use of ENDS and menthol cigarettes, which significantly decreased in PATH for males (1.7 to 0.9%) while remaining relatively constant for females (1.3 to 1.1%). Additionally, there were several sex differences in the prevalence of use patterns. Males had a higher prevalence of exclusive ENDS use than females in both PATH (2016/17: 5.4 vs. 4.7%) and NYTS (2017: 8.2 vs. 6.3%), although the differences were not significant. Males also had a higher prevalence of ENDS and non-menthol cigarette dual-use than females in PATH (2016/17: 1.3 vs. 0.6%), but not in NYTS (2017: 2.1 vs. 2.4%).

### 3.4. Prevalence Estimates by Race/Ethnicity

**Figure 1:**
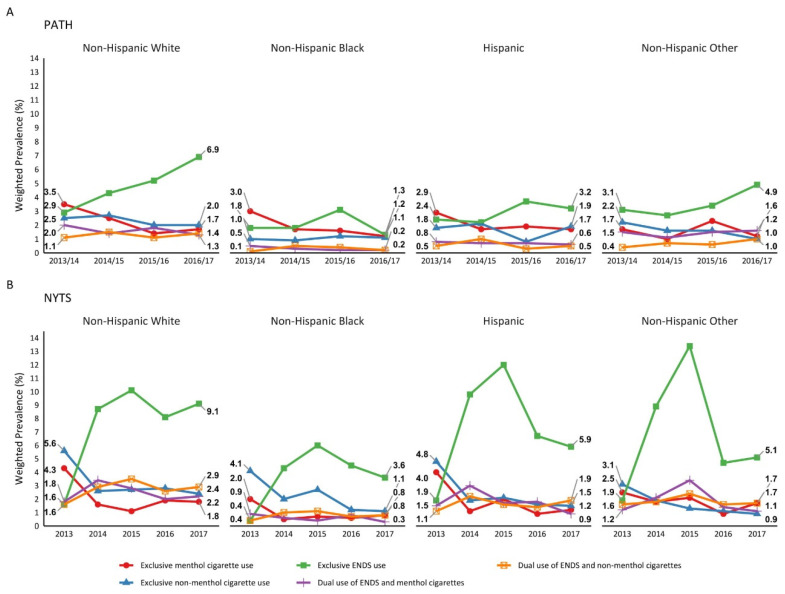
Prevalence of exclusive and dual use of ENDS and menthol/non-menthol cigarettes by race/ethnicity in (**A**) PATH and (**B**) NYTS.

Trends in ENDS and menthol/non-menthol cigarette use did not statistically differ by race/ethnicity, although there were differences in the magnitude of prevalence estimates across groups (Figure 1, Tables S5 and S6). In NYTS, from 2013 to 2017, exclusive non-menthol cigarette use decreased among all racial/ethnic groups and exclusive menthol cigarette use significantly decreased among Hispanic (4.0 to 1.2%) and NH White youth (4.3 to 1.8%). Also, in NYTS during the same period, exclusive ENDS use significantly increased for all racial/ethnic groups except NH Other. By 2017, NH White youth generally had a higher prevalence of all tobacco use patterns compared to other racial/ethnic groups in both surveys. Notably, compared to NH Black youth, NH White youth in NYTS had significantly higher prevalence of exclusive ENDS use (9.1 vs. 3.6%), dual ENDS and menthol cigarettes use (2.2 vs. 0.3%), and dual ENDS and non-menthol cigarettes use (2.9 vs. 0.8%).

### 3.5. Prevalence Estimates by Socioeconomic Status

**Figure 2:**
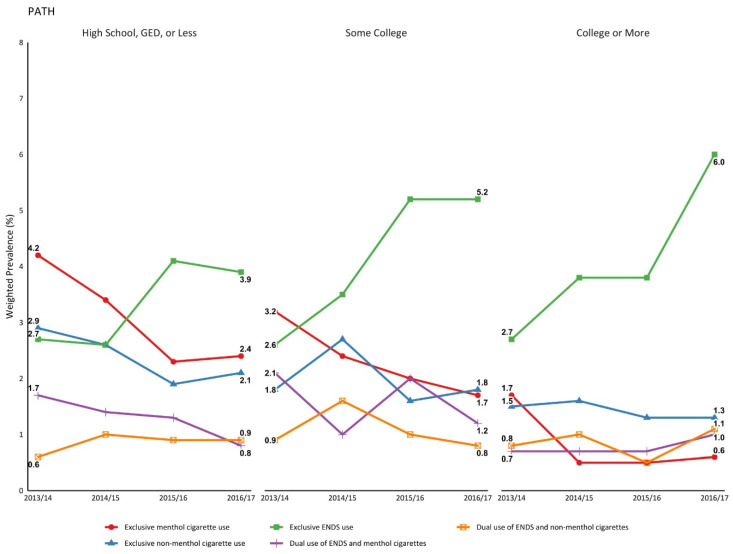
Prevalence of exclusive and dual use of ENDS and menthol/non-menthol cigarettes by parental education level in PATH.

Trends in ENDS and menthol/non-menthol cigarette use were relatively consistent by socioeconomic status, although the magnitude of prevalence estimates differed across groups. For youth whose parent had a higher education level (Table S7, Figure 2), higher total household income (Table S8, Figure S3), and homeownership (Table S9, Figure S4), the increase in exclusive ENDS use was significant. For instance, among youth whose parent had some college education, exclusive ENDS use increased from 2.6% in 2013/14 to 5.2% in 2016/17, while increasing from 2.7 to 6.0% if the parent had a college degree. Exclusive menthol cigarette use was the only other tobacco use pattern to significantly change over time, but it decreased regardless of the parent’s education level. Prevalence of use patterns by socioeconomic status varied widely by 2016/17. Among youth whose parents had higher education levels, higher total household income, and owned their own homes, the prevalence of exclusive ENDS use was higher than their respective counterparts. Lastly, exclusive menthol cigarette use was significantly lower if a parent had a college degree (0.6% vs. some college: 1.7% or high school: 2.4%), the total household income was $50,000 or more (0.8% vs. total household income less than $50,000: 2.6%), and if the home was owned (1.0% vs. home not owned: 2.7%).

## 4. Discussion

In this study, we examined sociodemographic differences in trends of current exclusive and dual use of ENDS and menthol and non-menthol cigarettes among 15–17-year-olds in the United States. Using two nationally representative surveys that have different modes of administration, PATH and NYTS, we found that exclusive ENDS use became the most prevalent use pattern by 2015 and was the only use pattern to significantly increase from 2013 to 2017 (PATH: 2.7 to 5.0%, NYTS: 1.5 to 7.3%). Both exclusive menthol and non-menthol cigarette use patterns continued to steadily decline during this time. Neither dual use of ENDS and menthol cigarettes nor dual use of ENDS and non-menthol cigarettes changed significantly in either survey.

We examined differences in tobacco use trends between males and females and found minimal differences as exclusive ENDS use increased, while exclusive menthol and non-menthol cigarette use decreased in both groups. The most notable difference by sex was that dual use of ENDS and menthol cigarettes decreased significantly among males (PATH: 1.7 to 0.9%) but not among females (PATH: 1.3 to 1.1%). There were also interesting differences in absolute prevalence estimates. Specifically, we found that exclusive ENDS use was significantly higher among males than females in PATH 2013/14 (3.4 vs. 1.9%), and while prevalence remained higher among males in 2016/17, the difference was no longer significant (5.4 vs. 4.7%). These findings build upon research compiled in a literature review examining studies on boy/girl differences in exclusive ENDS use among youth utilizing survey data from 2011 to 2016 [63]. Half the studies included in that review observed no differences, whereas half observed that boys were more likely to use ENDS than girls [63]. Several factors, including changes occurring in the marketplace, like the introduction of new device types and more appealing e-liquid flavors, and changes in marketing strategies, may be involved if the prevalence of exclusive ENDS use among females is truly rising. Future research will need to examine sex and gender differences in tobacco use patterns among youth in more detail to better understand the changing behaviors in these groups.

Regarding racial/ethnic differences, our results indicate similar trends of cigarette and ENDS use, but the prevalence of most tobacco use patterns was generally higher among NH White youth compared to other racial/ethnic groups. This finding is consistent with the results from previous studies examining single, dual-, and poly-tobacco use [64,65]. Furthermore, we found that NH White youth were significantly more likely to use ENDS, either exclusively or concurrently with menthol/non-menthol cigarettes, than NH Black youth. Past studies have shown that NH Black youth are less likely to initiate at an earlier age and have a lower prevalence of cigarette smoking than other racial/ethnic groups [66,67]. This appears to hold true for ENDS use as well [36], but we are likely missing other key racial/ethnic differences in tobacco use by only examining cigarette and ENDS use in this study. For instance, 9.2% (95% CI: 7.0–12.1) of NH Black youth in high school were past 30-day users of cigars in 2020 compared to 5.6% (95% CI: 3.8–8.2) of Hispanic youth and 4.2% (95% CI: 3.2–5.5) of NH White youth [35]. ENDS have played a major role in driving the increase in tobacco use among youth, but taking action by regulating ENDS alone without accounting for other tobacco products will not be sufficient and could exacerbate inequities.

We also examined differences in tobacco product use by indicators of socioeconomic status, and the findings are noteworthy. For instance, while the prevalence of exclusive menthol cigarette use decreased over time regardless of parental educational attainment, menthol cigarette use was significantly lower among youth whose parent had a college degree or higher (vs. high school or less or some college). Exclusive menthol cigarette use was also significantly lower among those living in households with higher annual income and owned homes compared to their respective counterparts. Furthermore, for youth whose parents had higher levels of education, higher total household income, and homeownership, the prevalence of other tobacco use patterns became less common as exclusive ENDS use became dominant. Different types of marketing may be playing a role in these findings. Youth with low socioeconomic status are exposed to more cigarette advertising [68,69], whereas youth with high socioeconomic status may be exposed to more ENDS advertising [69]. Exposure to ENDS marketing is associated with ENDS use among youth [70,71] and the relationship between socioeconomic status and ENDS use is mediated by ENDS advertisement [72]. Targeting youth tobacco control policies by socioeconomic status might be a more effective strategy to reduce youth tobacco use for all products.

Prevalence trends in dual-use patterns did not change significantly in either survey and did not change among any sociodemographic subgroups, except for a decrease in dual use of ENDS and menthol cigarettes among males in PATH. This is likely a consequence of the opposing trends in ENDS and cigarette use, which might be counteracting each other as far as dual-use is concerned. Nonetheless, changes in dual-use should continue to be monitored, with a focus on specific use patterns and sociodemographic groups. Though not significant, our results show that from 2013 to 2017 in NYTS, dual use of ENDS and non-menthol cigarettes increased from 1.0 to 2.4% among females (vs. 1.7 to 2.1% among males), 1.1 to 2.0% among Hispanic youth, and 1.6 to 2.9% among NH White youth. A sustained increase is concerning as dual users are at greater risk for engaging in risky behaviors like alcohol and illicit drug use compared to exclusive cigarette or ENDS users [73]. Furthermore, youth dual users are likely to remain dual users and more likely to continue using cigarettes than to transition to exclusive ENDS use or no use at all [74]. In particular, depending on whether they use cigarettes and/or ENDS daily or almost daily, youth dual-users have higher odds of nicotine dependence and lower or similar intentions of quitting compared to exclusive cigarette smokers [75]. Assessing the frequency of product use and sociodemographic characteristics of dual-use is essential to better understand the consequences of youth dual and polytobacco use.

Over many years, banning menthol cigarettes has been proposed to further reduce overall tobacco use among youth and address tobacco-related health disparities. The FDA announced in April 2021 its intention to ban menthol in cigarettes and all flavors in cigars [15], but it may still take a few years to be enacted. Examining cigarette use, a study found that the FDA’s previous ban on flavored cigarette products, included in the 2009 Family Smoking Prevention and Tobacco Control Act, was effective in reducing the odds of cigarette use among youth ages 12 to 17 by 43% compared to models predicting the probability of cigarette use without the ban [76]. An earlier study also reported that the probability of youth using any tobacco product decreased by 6.1% after the ban, but the probability of using at least one non-cigarette tobacco product (i.e., cigars, smokeless tobacco, or pipes) increased by 14.2% [77]. Both studies seemed to indicate that at least some proportion of youth who used flavored cigarettes chose to use menthol cigarettes after the ban [76,77]; other youth likely substituted flavored cigarettes with legally flavored tobacco products [77]. Due to limitations in data before the ban, neither the impact of hookah nor ENDS could be analyzed in these studies [76,77]. Our findings suggest that a menthol cigarette ban would have the greatest impact on youth with lower socioeconomic status, particularly if combined with a ban on all flavored cigars.

It is important to note that the prevalence estimates found in NYTS were higher than the prevalence estimates found in PATH. Furthermore, while in PATH the prevalence of exclusive menthol cigarette use was higher or similar to exclusive non-menthol cigarette use, in NYTS we observed the opposite. Additionally, the prevalence of dual-use patterns in NYTS were similar to the prevalence of exclusive cigarette use patterns, whereas dual-use patterns were generally lower than all exclusive use patterns in PATH. Some of these differences could be due to differences in data collection setting and methodology: PATH is a household-based survey conducted in-home with ACASI [54] and NYTS is a school-based survey conducted in the classroom using pencil and paper [56,57,58,59,60]. School-based surveys tend to report higher prevalence estimates of tobacco use than household-based surveys [78], even when methods like ACASI are used to maximize privacy [79]. At home, youth may be concerned that their responses might be disclosed to their parents, while at school, youth may be influenced by their peers; it is difficult to know which estimates are more representative of the overall youth tobacco use patterns in the US [80]. Despite these differences, the trends across the two surveys were consistent, suggesting a general downward trend in menthol and non-menthol cigarette use regardless of the modality of the survey interview.

There are several limitations in this study. First, we examined data from 2013 to 2017 because it allowed for better comparability of trends over time for these two surveys. In doing so, we were unable to examine ENDS use classified by menthol and non-menthol flavoring, as data on these measures only became available starting in PATH 2014/15 and NYTS 2016. Furthermore, the years we examined do not fully capture the more recent increases in ENDS use due to the emergence of JUUL and pod-based systems [81,82]. Newer PATH and NYTS data are now available, but only data from 2013/14 and 2016/17 are truly nationally representative in the PATH study. There is attrition in the number of completed interviews by 15–17-year-olds in PATH 2014/15 and PATH 2015/16 that could be attributed to various factors, including nonresponse and more youth aging up into adults than 12–14-year-old youth aging up into the 15–17-year-old category [55]. Weights for PATH 2014/15 and PATH 2015/16 account for attrition and allow for pseudo-cross-sectional analyses, but since they only follow the cohort from PATH 2013/14, there is a possibility that the weighted estimates from these waves are not nationally representative at the time these waves were conducted. Future analyses using more recent nationally representative data are needed to better understand trends in the post-JUUL era. Second, we utilized an inclusive definition of current product use (i.e., product use on at least 1 day in the past 30 days) that is commonly used in studies examining tobacco use among youth [36,83]. This definition made our estimates easily comparable to other studies and allowed us sufficiently large sample sizes to assess differences across sociodemographic subgroups. However, this definition does not distinguish between experimental and established users. Most experimental users will not become established users, which means our current use prevalence estimates are likely overestimating tobacco behaviors that will result in long-term use. Future research would benefit from examining more stringent definitions of tobacco use, focusing on frequent users or established users. Third, in our definition of menthol and non-menthol cigarette use, we classified participants who were missing information about menthol-flavored cigarettes with participants who used non-menthol cigarettes. While it is possible that we overestimated the prevalence of current non-menthol cigarette use and underestimated the prevalence of current menthol cigarette use among youth, we were able to preserve our estimates of overall cigarette use prevalence. Fourth, only PATH contained information about socioeconomic status—we were not able to examine differences in use patterns with NYTS. Fifth, youth tobacco users are more likely to be polytobacco users [65], especially youth ENDS users [84,85]. While this study only examined ENDS and cigarette use, future studies may want to consider examining other tobacco products popular among youth when assessing these trends. Finally, our results are based on cross-sectional data (NYTS) or cross-sectional analysis of longitudinal data in which observations are not independent (PATH). Changes in prevalence do not capture within-individual changes in tobacco product use over time. To directly answer this question, transition analyses on prospective longitudinal data are needed.

Despite these limitations, this study provides short-term trends in the prevalence of exclusive menthol/non-menthol cigarette use, exclusive ENDS use, and dual cigarette and ENDS use among youth in the US. By comparing the results from two nationally representative youth surveys with different modes of administration, we provide evidence that the prevalence of cigarette use continued to decline between 2013 and 2017 while the prevalence of ENDS use dramatically increased. Our study also indicates that if the proposed menthol ban is put into effect by the FDA, youth with low socioeconomic status are the most likely to be impacted. Additionally, interventions and prevention strategies aiming to reduce exclusive and dual-use of ENDS should target NH White youth and youth with high socioeconomic status.

## 5. Conclusions

Data from two nationally representative surveys showed a rise in exclusive ENDS use and a steady decline in both exclusive menthol cigarette and exclusive non-menthol cigarette use among 15–17-year-olds from 2013 to 2017. While dual-use patterns did not change significantly across the years examined, the slight upward trend of ENDS and non-menthol cigarette use is concerning. Exclusive and dual-use of menthol and non-menthol cigarettes with ENDS also varied by sex, race/ethnicity, parental education level, annual household income, and homeownership. NH White youth and youth with high socioeconomic status, in particular, were more likely to use ENDS than other racial/ethnic groups and youth with low socioeconomic status, respectively. Continual monitoring of these tobacco use patterns will be essential for understanding changes in use patterns by sociodemographic characteristics and informing future prevention strategies targeting tobacco use among this age group.

## Figures and Tables

**Table 1:** Sociodemographic characteristics and overall ENDS and cigarette use patterns of 15–17-year-olds in 2013 and 2017. Population Assessment of Tobacco and Health (PATH) and National Youth Tobacco Survey (NYTS).

	PATH				NYTS			
	Wave 1 (2013/14) *n* = 6596		Wave 4 (2016/17) *n* = 7414		2013 *n* = 7363		2017 *n* = 7582	
	*n*	Weighted % (95% CI)	*n*	Weighted % (95% CI)	*n*	Weighted % (95% CI)	*n*	Weighted % (95% CI)
**Sex**								
Female	3237	48.8 (48.7, 48.9)	3586	49.0 (48.9, 49.1)	3795	49.4 (47.0, 51.7)	3785	49.2 (47.0, 51.3)
Male	3350	51.2 (51.1, 51.3)	3804	51.0 (50.9, 51.1)	3567	50.7 (48.3, 53.0)	3754	50.8 (48.7, 53.0)
**Race/Ethnicity**								
Hispanic	1828	21.9 (21.7, 22.0)	2238	23.2 (23.0, 23.3)	1734	21.3 (17.0, 26.4)	1954	23.2 (18.6, 28.6)
Non-Hispanic White	3229	55.5 (55.3, 55.7)	3326	53.5 (53.3, 53.8)	3216	53.6 (47.4, 59.7)	3238	53.4 (47.0, 59.7)
Non-Hispanic Black	891	13.6 (13.4, 13.8)	1004	13.6 (13.4, 13.8)	1254	13.9 (10.8, 17.8)	1300	12.4 (9.9, 15.6)
Non-Hispanic Other	578	9.1 (8.9, 9.2)	668	9.7 (9.6, 9.9)	955	11.2 (8.9, 13.9)	847	11.0 (8.8, 13.7)
**Parental Education Level**								
High school diploma, GED, or less	2633	36.4 (34.4, 38.4)	2831	34.7 (33.0, 36.4)	NA		NA	
Some college	2126	32.9 (31.2, 34.5)	2314	31.1 (29.6, 32.5)	NA		NA	
College or more	1780	30.7 (28.4, 33.1)	2188	34.3 (32.3, 36.3)	NA		NA	
**Annual Household Income**								
Less than $50,000	NA		3465	43.6 (41.9, 45.2)	NA		NA	
$50,000 or more	NA		3722	56.4 (54.8, 58.1)	NA		NA	
**Homeownership**								
Home not owned	NA		2938	36.7 (35.2, 38.2)	NA		NA	
Home owned	NA		4397	63.3 (61.8, 64.8)	NA		NA	
**Tobacco Use Pattern**								
Never/non-current	5927	89.8 (89.0, 90.6)	6682	89.7 (88.7, 90.5)	6428	87.1 (85.6, 88.5)	6566	85.5 (83.0, 87.7)
Exclusive menthol cigarette use	212	3.1 (2.7, 3.6)	121	1.6 (1.3, 1.9)	292	3.7 (3.0, 4.5)	117	1.5 (1.0, 2.2)
Exclusive non-menthol cigarette use	139	2.1 (1.8, 2.5)	135	1.8 (1.5, 2.1)	344	4.9 (4.3, 5.7)	149	1.9 (1.5, 2.4)
Exclusive ENDS use	163	2.7 (2.2, 3.2)	336	5.0 (4.4, 5.7)	100	1.5 (1.2, 1.9)	486	7.3 (5.8, 9.0)
Dual use of ENDS + menthol cigarettes	105	1.5 (1.2, 1.8)	76	1.0 (0.8, 1.3)	115	1.5 (1.1, 2.1)	108	1.6 (1.2, 2.1)
Dual use of ENDS + non-menthol cigarettes	50	0.8 (0.6, 1.0)	64	1.0 (0.7, 1.3)	84	1.3 (0.1, 1.8)	156	2.3 (1.7, 3.1)

Abbreviations: (95% CI) = 95% confidence interval; Missing (i.e., cigarette and/or ENDS use not ascertained): PATH 2013/14 (*n* = 55), PATH 2016/17 (*n* = 42), NYTS 2013 (*n* = 392), NYTS 2017 (*n* = 155); Questions about annual household income and homeownership were not asked in PATH Wave 1 (2013/14). Socioeconomic status questions (parental education level, annual household income, and homeownership) were not asked in NYTS.

## Data Availability

Publicly available datasets were analyzed in this study. PATH data presented in this study are openly available by ICPSR at https://doi.org/10.3886/ICPSR36498.v11 (accessed on 27 May 2021), and NYTS data are available by the CDC at https://www.cdc.gov/tobacco/data_statistics/surveys/nyts/index.htm (accessed on 27 May 2021).

## Supplementary Materials

The following are available online https://www.mdpi.com/article/10.3390/ijerph18157781/s1—Table S1: Sociodemographic characteristics and overall exclusive and dual use patterns of ENDS and menthol/non-menthol cigarettes for 15–17-year-olds in the Population Assessment of Tobacco and Health (PATH), 2013–2017, Table S2: Sociodemographic characteristics and overall exclusive and dual use patterns of ENDS and menthol/non-menthol cigarettes for 15–17-year-olds in the National Youth Tobacco Survey (NYTS), 2013–2017, Table S3: Patterns of exclusive and dual use of ENDS and menthol/non-menthol cigarettes among 15–17-year-olds by sex in the Population Assessment of Tobacco and Health (PATH), 2013–2017, Table S4: Patterns of exclusive and dual use of ENDS and menthol/non-menthol cigarettes among 15–17-year-olds by sex in the National Youth Tobacco Survey (NYTS), 2013–2017, Table S5: Patterns of exclusive and dual use of ENDS and menthol/non-menthol cigarettes among 15–17-year-olds by race/ethnicity in the Population Assessment of Tobacco and Health (PATH), 2013–2017, Table S6: Patterns of exclusive and dual use of ENDS and menthol/non-menthol cigarettes among 15–17-year-olds by race/ethnicity in the National Youth Tobacco Survey (NYTS), 2013–2017, Table S7: Patterns of exclusive and dual use of ENDS and menthol/non-menthol cigarettes among 15–17-year-olds by parental education level in the Population Assessment of Tobacco and Health (PATH), 2013–2017, Table S8: Patterns of exclusive and dual use of ENDS and menthol/non-menthol cigarettes among 15–17-year-olds by annual household income in the Population Assessment of Tobacco and Health (PATH), 2014–2017, Table S9: Patterns of exclusive and dual use of ENDS and menthol/non-menthol cigarettes among 15–17-year-olds by homeownership status in the Population Assessment of Tobacco and Health (PATH), 2014–2017, Figure S1: Prevalence of exclusive and dual use of ENDS and menthol/non-menthol cigarettes overall, Figure S2: Prevalence of exclusive and dual use of ENDS and menthol/non-menthol cigarettes by sex, Figure S3: Prevalence of exclusive and dual use of ENDS and menthol/non-menthol cigarettes overall by household income (PATH only), and Figure S4: Prevalence of exclusive and dual use of ENDS and menthol/non-menthol cigarettes overall by homeownership (PATH only).
