# Supplementary material for: Sociodemographic Patterns of Exclusive and Dual Use of ENDS and Menthol/Non-Menthol Cigarettes among US Youth (Ages 15–17) Using Two Nationally Representative Surveys (2013–2017)
Source: Int J Environ Res Public Health. 2021 Jul 22;18(15):7781. doi: 10.3390/ijerph18157781 (PMC8345686; doi:10.3390/ijerph18157781)
Supplement: Supplementary file 1 [file ijerph-18-07781-s001.zip › ijerph-1281577-supplementary.pdf]

Table S1: Sociodemographic characteristics and overall exclusive and dual use patterns of ENDS and menthol/non-menthol cigarettes for 15-17-year-olds in the Population Assessment of Tobacco and Health (PATH), 2013-2017

	PATH							
	Wave 1 (2013/14) n = 6596		Wave 2 (2014/15) n = 5858		Wave 3 (2015/16) n = 5782		Wave 4 (2016/17) n = 7414	
	n	weighted % (95% CI)	n	weighted % (95% CI)	n	weighted % (95% CI)	n	weighted % (95% CI)
**Sex**								
Female	3237	48.8 (48.7, 48.9)	2848	48.7 (48.2, 49.1)	2754	48.4 (48.0, 48.8)	3586	49.0 (48.9, 49.1)
Male	3350	51.2 (51.1, 51.3)	3003	51.3 (50.9, 51.8)	3020	51.6 (51.2, 52.0)	3804	51.0 (50.9, 51.1)
**Race/Ethnicity**								
Hispanic	1828	21.9 (21.7, 22.0)	1688	22.3 (22.0, 22.6)	1722	22.8 (22.4, 23.2)	2238	23.2 (23.0, 23.3)
Non-Hispanic White	3229	55.5 (55.3, 55.7)	2797	55.2 (54.7, 55.7)	2678	54.9 (54.4, 55.4)	3326	53.5 (53.3, 53.8)
Non-Hispanic Black	891	13.6 (13.4, 13.8)	760	13.4 (13.0, 13.8)	741	13.3 (13.0, 13.6)	1004	13.6 (13.4, 13.8)
Non-Hispanic Other	578	9.1 (8.9, 9.2)	533	9.1 (8.8, 9.5)	524	9.0 (8.6, 9.4)	668	9.7 (9.6, 9.9)
**Parental Education Level**								
High school diploma, GED, or less	2633	36.4 (34.4, 38.4)	2117	34.5 (32.9, 36.1)	2169	33.8 (31.8, 35.8)	2831	34.7 (33.0, 36.4)
Some college	2126	32.9 (31.2, 34.5)	1793	32.5 (30.6, 34.4)	1829	32.2 (30.6, 33.9)	2314	31.1 (29.6, 32.5)
College or more	1780	30.7 (28.4, 33.1)	1611	33.0 (30.8, 35.4)	1676	34.0 (31.8, 36.3)	2188	34.3 (32.3, 36.3)
**Annual Household Income**								
Less than $50,000		NA	2695	45.8 (43.8, 47.9)	2729	44.4 (42.4, 46.4)	3465	43.6 (41.9, 45.2)
$50,000 or more		NA	2677	54.2 (52.1, 56.2)	2813	55.6 (53.6, 57.6)	3722	56.4 (54.8, 58.1)
**Homeownership**								
Home not owned		NA	2188	36.8 (35.1, 38.6)	2281	36.9 (35.0, 38.8)	2938	36.7 (35.2, 38.2)
Home owned		NA	3322	63.2 (61.4, 64.9)	3394	63.1 (61.2, 65.0)	4397	63.3 (61.8, 64.8)
**Tobacco Use Pattern**								
Never / non-current	5927	89.8 (89.0, 90.6)	5295	90.1 (89.2, 91.0)	5250	90.4 (89.4, 91.2)	6682	89.7 (88.7, 90.5)
Exclusive menthol cigarette use	212	3.1 (2.7, 3.6)	119	2.1 (1.7, 2.5)	99	1.6 (1.3, 2.0)	121	1.6 (1.3, 1.9)
Exclusive non-menthol cigarette use	139	2.1 (1.8, 2.5)	139	2.3 (1.8, 2.8)	85	1.6 (1.3, 2.0)	135	1.8 (1.5, 2.1)
Exclusive ENDS use	163	2.7 (2.2, 3.2)	174	3.3 (2.8, 3.9)	234	4.3 (3.7, 5.1)	336	5.0 (4.4, 5.7)
Dual use of ENDS + menthol cigarettes	105	1.5 (1.2, 1.8)	64	1.1 (0.8, 1.4)	71	1.3 (1.0, 1.8)	76	1.0 (0.8, 1.3)
Dual use of ENDS + non-menthol cigarettes	50	0.8 (0.6, 1.0)	67	1.2 (0.9, 1.5)	43	0.8 (0.6, 1.1)	64	1.0 (0.7, 1.3)

Abbreviations: (95% CI) = 95% Confidence Interval

Missing (i.e. cigarette and/or ENDS use not ascertained): PATH Wave 1 (n=55), PATH Wave 2 (n=48), PATH Wave 3 (n=30), PATH Wave 4 (n=42)

Questions about annual household income and homeownership were not asked in PATH Wave 1 (2013/14).

Table S2: Sociodemographic characteristics and overall exclusive and dual use patterns of ENDS and menthol/non-menthol cigarettes for 15-17-year-olds in the National Youth Tobacco Survey (NYTS), 2013-2017

	NYTS									
	2013 n =7363		2014 n = 8460		2015 n =7088		2016 n =8095		2017 n =7582	
	n	weighted % (95% CI)	n	weighted % (95% CI)	n	weighted % (95% CI)	n	weighted % (95% CI)	n	weighted % (95% CI)
**Sex**										
Female	3795	49.4 (47.0, 51.7)	4111	50.3 (47.0, 53.5)	3443	49.4 (46.2, 52.5)	4029	50.4 (48.5, 52.4)	3785	49.2 (47.0, 51.3)
Male	3567	50.7 (48.3, 53.0)	4315	49.7 (46.5, 53.0)	3618	50.7 (47.5, 53.8)	4040	49.6 (47.6, 51.5)	3754	50.8 (48.7, 53.0)
**Race/Ethnicity**										
Hispanic	1734	21.3 (17.0, 26.4)	2241	20.9 (17.5, 24.8)	1978	22.9 (18.4, 28.2)	2298	24.7 (20.8, 29.2)	1954	23.2 (18.6, 28.6)
Non-Hispanic White	3216	53.6 (47.4, 59.7)	3607	54.2 (48.9, 59.4)	3327	54.8 (47.7, 61.7)	3283	53.2 (47.7, 58.7)	3238	53.4 (47.0, 59.7)
Non-Hispanic Black	1254	13.9 (10.8, 17.8)	1294	15.0 (11.1, 20.0)	973	13.4 (10.0, 17.9)	1268	12.3 (9.7, 15.4)	1300	12.4 (9.9, 15.6)
Non-Hispanic Other	955	11.2 (8.9, 13.9)	1043	10.0 (8.4, 11.8)	593	8.9 (6.6, 11.9)	994	9.8 (8.3, 11.5)	847	11.0 (8.8, 13.7)
**Parental Education Level**										
High school diploma, GED, or less	NA		NA		NA		NA		NA	
Some college	NA		NA		NA		NA		NA	
College or more	NA		NA		NA		NA		NA	
**Annual Household Income**										
Less than $50,000	NA		NA		NA		NA		NA	
$50,000 or more	NA		NA		NA		NA		NA	
**Homeownership**										
Home not owned	NA		NA		NA		NA		NA	
Home owned	NA		NA		NA		NA		NA	
**Tobacco Use Pattern**										
Never / non-current	6428	87.1 (85.6, 88.5)	6990	83.0 (80.6, 85.2)	5763	80.9 (78.7, 83.0)	7064	85.9 (83.9, 87.6)	6566	85.5 (83.0, 87.7)
Exclusive menthol cigarette use	292	3.7 (3.0, 4.5)	135	1.3 (1.0, 1.9)	107	1.4 (1.1, 1.8)	108	1.5 (1.0, 2.1)	117	1.5 (1.0, 2.2)
Exclusive non-menthol cigarette use	344	4.9 (4.3, 5.7)	213	2.3 (1.9, 2.8)	156	2.4 (1.7, 3.3)	150	2.2 (1.7, 2.8)	149	1.9 (1.5, 2.4)
Exclusive ENDS use	100	1.5 (1.2, 1.9)	693	8.2 (6.7, 10.1)	717	10.3 (8.9, 11.8)	507	6.9 (5.9, 8.0)	486	7.3 (5.8, 9.0)
Dual use of ENDS + menthol cigarettes	115	1.5 (1.1, 2.1)	229	2.7 (2.2, 3.4)	163	2.3 (1.8, 3.0)	130	1.7 (1.4, 2.1)	108	1.6 (1.2, 2.1)
Dual use of ENDS + non-menthol cigarettes	84	1.3 (0.1, 1.8)	200	2.4 (1.9, 3.0)	182	2.7 (2.0, 3.4)	136	2.0 (1.5, 2.5)	156	2.3 (1.7, 3.1)

Abbreviations: (95% CI) = 95% Confidence Interval

Missing (i.e. cigarette and/or ENDS use not ascertained): NYTS 2013 (n=392), NYTS 2014 (n=453), NYTS 2015 (n=133), NYTS 2016 (n=395), NYTS 2017 (n=155)

Socioeconomic status questions (parental education level, annual household income, and homeownership) were not asked in NYTS.

Table S3: Patterns of exclusive and dual use of ENDS and menthol/non-menthol cigarettes among 15-17-year-olds by sex in the Population Assessment of Tobacco and Health (PATH), 2013-2017

	PATH							
	Wave 1 (2013/14) n = 6587		Wave 2 (2014/15) n = 5851		Wave 3 (2015/16) n = 5774		Wave 4 (2016/17) n = 7390	
	n	weighted % (95% CI)	n	weighted % (95% CI)	n	weighted % (95% CI)	n	weighted % (95% CI)
**Female**								
Never / non-current	2941	90.8 (89.8, 91.8)	2585	90.6 (89.3, 91.8)	2494	90.3 (88.9, 91.6)	3260	90.3 (89.1, 91.4)
Exclusive menthol cigarette use	111	3.5 (2.8, 4.3)	60	2.1 (1.6, 2.7)	54	1.8 (1.4, 2.4)	60	1.6 (1.3, 2.1)
Exclusive non-menthol cigarette use	65	1.9 (1.5, 2.4)	71	2.2 (1.7, 3.0)	48	1.9 (1.4, 2.5)	57	1.6 (1.2, 2.1)
Exclusive ENDS use	57	1.9 (1.5, 2.5)	69	2.7 (2.1, 3.5)	105	4.0 (3.1, 5.0)	147	4.7 (4.0, 5.5)
Dual use of ENDS + menthol cigarettes	45	1.3 (0.9, 1.8)	36	1.3 (1.0, 1.8)	32	1.2 (0.8, 1.8)	41	1.1 (0.8, 1.6)
Dual use of ENDS + non-menthol cigarettes	18	0.6 (0.3, 1.0)	27	1.0 (0.6, 1.5)	21	0.8 (0.5, 1.3)	21	0.6 (0.4, 1.0)
**Male**								
Never / non-current	2977	88.9 (87.7, 89.9)	2704	89.7 (88.4, 90.9)	2749	90.5 (89.2, 91.6)	3399	89.0 (87.7, 90.1)
Exclusive menthol cigarette use	101	2.8 (2.3, 3.5)	59	2.0 (1.5, 2.6)	45	1.4 (1.0, 1.9)	60	1.5 (1.1, 1.9)
Exclusive non-menthol cigarette use	74	2.3 (1.8, 2.9)	68	2.3 (1.7, 3.0)	37	1.3 (0.9, 1.8)	78	1.9 (1.6, 2.4)
Exclusive ENDS use	106	3.4 (2.7, 4.2)	104	3.8 (3.1, 4.6)	128	4.6 (3.8, 5.6)	189	5.4 (4.5, 6.4)
Dual use of ENDS + menthol cigarettes	60	1.7 (1.3, 2.2)	28	0.9 (0.6, 1.3)	39	1.4 (1.0, 1.9)	35	0.9 (0.7, 1.2)
Dual use of ENDS + non-menthol cigarettes	32	0.9 (0.7, 1.3)	40	1.4 (1.0, 1.9)	22	0.8 (0.5, 1.3)	43	1.3 (1.0, 1.8)

Abbreviations: (95% CI) = 95% Confidence Interval

Table S4: Patterns of exclusive and dual use of ENDS and menthol/non-menthol cigarettes among 15-17-year-olds by sex in the National Youth Tobacco Survey (NYTS), 2013-2017

	NYTS									
	2013 n =7362		2014 n = 8426		2015 n =7061		2016 n =8069		2017 n =7539	
	n	weighted % (95% CI)	n	weighted % (95% CI)	n	weighted % (95% CI)	n	weighted % (95% CI)	n	weighted % (95% CI)
**Female**										
Never / non-current	3360	88.5 (86.8, 90.1)	3520	85.4 (83.1, 87.4)	2883	83.5 (81.1, 85.5)	3589	87.7 (85.3, 89.7)	3311	85.9 (83.3, 88.2)
Exclusive menthol cigarette use	151	3.8 (2.9, 4.8)	58	1.2 (0.9, 1.8)	61	1.7 (1.2, 2.3)	62	1.7 (1.1, 2.5)	66	1.9 (1.2, 2.9)
Exclusive non-menthol cigarette use	164	4.4 (3.6, 5.4)	83	2.0 (1.5, 2.7)	69	2.0 (1.5, 2.8)	69	1.9 (1.4, 2.6)	85	2.0 (1.6, 2.6)
Exclusive ENDS use	37	1.1 (0.7, 1.7)	283	6.9 (5.4, 8.7)	300	8.8 (7.4, 10.4)	210	5.7 (4.7, 6.9)	210	6.3 (4.9, 8.1)
Dual use of ENDS + menthol cigarettes	51	1.3 (0.9, 1.8)	89	2.4 (1.9, 3.1)	65	1.9 (1.3, 2.6)	47	1.4 (0.9, 2.1)	44	1.5 (0.9, 2.4)
Dual use of ENDS + non-menthol cigarettes	32	1.0 (0.6, 1.7)	78	2.1 (1.5, 3.1)	65	2.2 (1.5, 3.2)	52	1.7 (1.1, 2.5)	69	2.4 (1.6, 3.6)
**Male**										
Never / non-current	3067	85.7 (83.6, 87.5)	3445	80.7 (77.5, 83.5)	2859	78.5 (75.6, 81.1)	3453	84.0 (81.9, 86.0)	3216	85.2 (82.3, 87.6)
Exclusive menthol cigarette use	141	3.6 (2.7, 4.6)	76	1.4 (0.9, 2.2)	45	1.1 (0.8, 1.6)	46	1.2 (0.8, 1.9)	51	1.1 (0.7, 1.7)
Exclusive non-menthol cigarette use	180	5.5 (4.6, 6.5)	130	2.7 (2.0, 3.5)	86	2.8 (1.8, 4.2)	81	2.4 (1.8, 3.2)	64	1.8 (1.3, 2.6)
Exclusive ENDS use	63	1.9 (1.3, 2.6)	404	9.5 (7.7, 11.7)	415	11.8 (9.9, 14.0)	295	8.0 (6.7, 9.4)	275	8.2 (6.4, 10.5)
Dual use of ENDS + menthol cigarettes	64	1.8 (1.1, 2.8)	139	3.1 (2.3, 4.1)	98	2.8 (2.2, 3.7)	82	2.1 (1.7, 2.6)	63	1.6 (1.1, 2.2)
Dual use of ENDS + non-menthol cigarettes	52	1.7 (1.2, 2.3)	121	2.7 (2.0, 3.5)	115	3.1 (2.3, 4.0)	83	2.2 (1.7, 3.0)	85	2.1 (1.6, 2.9)

Abbreviations: (95% CI) = 95% Confidence Interval

Table S5: Patterns of exclusive and dual use of ENDS and menthol/non-menthol cigarettes among 15-17-year-olds by race/ethnicity in the Population Assessment of Tobacco and Health (PATH), 2013-2017

	PATH							
	Wave 1 (2013/14) n = 6526		Wave 2 (2014/15) n = 5778		Wave 3 (2015/16) n = 5665		Wave 4 (2016/17) n = 7236	
	n	weighted % (95% CI)	n	weighted % (95% CI)	n	weighted % (95% CI)	n	weighted % (95% CI)
**Hispanics**								
Never / non-current	1684	91.6 (90.1, 93.0)	1560	92.3 (90.6, 93.7)	1599	92.6 (91.2, 93.7)	2073	92.2 (90.7, 93.4)
Exclusive menthol cigarette use	51	2.9 (2.1, 3.9)	28	1.7 (1.1, 2.7)	32	1.9 (1.4, 2.7)	38	1.7 (1.2, 2.4)
Exclusive non-menthol cigarette use	29	1.8 (1.3, 2.5)	37	2.1 (1.4, 3.1)	13	0.8 (0.5, 1.3)	38	1.9 (1.4, 2.4)
Exclusive ENDS use	40	2.4 (1.7, 3.4)	35	2.2 (1.6, 3.1)	58	3.7 (2.8, 4.7)	63	3.2 (2.4, 4.2)
Dual use of ENDS + menthol cigarettes	17	0.8 (0.5, 1.4)	12	0.7 (0.4, 1.3)	14	0.7 (0.4, 1.4)	15	0.6 (0.3, 1.1)
Dual use of ENDS + non-menthol cigarettes	7	0.5 (0.2, 1.0)	16	1.0 (0.6, 1.6)	6	0.3 (0.1, 0.8)	11	0.5 (0.3, 1.0)
**Non-Hispanic White**								
Never / non-current	2831	88.0 (86.7, 89.2)	2452	87.7 (86.3, 89.0)	2385	88.5 (87.0, 89.9)	2890	86.8 (85.3, 88.2)
Exclusive menthol cigarette use	117	3.5 (2.8, 4.3)	68	2.5 (1.8, 3.3)	38	1.4 (1.0, 2.0)	57	1.7 (1.3, 2.2)
Exclusive non-menthol cigarette use	85	2.5 (2.0, 3.0)	80	2.7 (2.1, 3.5)	51	2.0 (1.5, 2.7)	69	2.0 (1.6, 2.5)
Exclusive ENDS use	89	2.9 (2.3, 3.7)	116	4.3 (3.4, 5.2)	131	5.2 (4.2, 6.4)	220	6.9 (5.9, 8.0)
Dual use of ENDS + menthol cigarettes	69	2.0 (1.6, 2.5)	38	1.4 (1.0, 2.0)	44	1.8 (1.2, 2.6)	44	1.3 (0.9, 1.9)
Dual use of ENDS + non-menthol cigarettes	38	1.1 (0.8, 1.6)	43	1.5 (1.0, 2.0)	29	1.1 (0.7, 1.7)	46	1.4 (1.0, 1.8)
**Non-Hispanic Black**								
Never / non-current	837	93.5 (91.9, 94.9)	724	94.7 (92.5, 96.3)	691	93.4 (91.0, 95.2)	963	95.9 (94.0, 97.3)
Exclusive menthol cigarette use	25	3.0 (2.1, 4.4)	12	1.7 (0.8, 3.5)	13	1.6 (0.9, 2.8)	13	1.2 (0.6, 2.4)
Exclusive non-menthol cigarette use	9	1.0 (0.5, 2.0)	9	0.9 (0.5, 1.8)	11	1.2 (0.6, 2.4)	13	1.1 (0.5, 2.4)
Exclusive ENDS use	14	1.8 (1.1, 3.0)	9	1.8 (0.8, 3.8)	21	3.1 (2.0, 4.8)	11	1.3 (0.7, 2.5)
Dual use of ENDS + menthol cigarettes	5	0.5 (0.2, 1.4)	3	0.3 (0.1, 1.1)	2	0.2 (0.1, 1.0)	2	0.2 (0.0, 0.7)
Dual use of ENDS + non-menthol cigarettes	1	0.1 (0.0, 0.7)	3	0.5 (0.2, 1.7)	3	0.4 (0.1, 1.4)	2	0.2 (0.1, 1.1)
**Non-Hispanic Other**								
Never / non-current	514	91.2 (88.8, 93.1)	487	92.9 (90.3, 94.8)	464	90.6 (88.1, 92.6)	594	90.3 (87.7, 92.3)
Exclusive menthol cigarette use	16	1.7 (1.1, 2.6)	9	1.0 (0.5, 1.9)	16	2.3 (1.3, 3.9)	10	1.2 (0.6, 2.2)
Exclusive non-menthol cigarette use	14	2.2 (1.3, 3.8)	10	1.6 (0.8, 3.2)	8	1.6 (0.7, 3.4)	9	1.0 (0.5, 2.1)
Exclusive ENDS use	18	3.1 (1.9, 4.9)	14	2.7 (1.4, 5.0)	22	3.4 (2.1, 5.3)	35	4.9 (3.3, 7.4)
Dual use of ENDS + menthol cigarettes	13	1.5 (0.8, 2.6)	9	1.1 (0.5, 2.3)	10	1.5 (0.7, 3.1)	15	1.6 (0.9, 2.9)
Dual use of ENDS + non-menthol cigarettes	3	0.4 (0.1, 1.3)	4	0.7 (0.2, 2.3)	4	0.6 (0.2, 1.7)	5	1.0 (0.3, 2.9)

Abbreviations: (95% CI) = 95% Confidence Interval

Table S6: Patterns of exclusive and dual use of ENDS and menthol/non-menthol cigarettes among 15-17-year-olds by race/ethnicity in the National Youth Tobacco Survey (NYTS), 2013-2017

	NYTS									
	2013 n =7159		2014 n = 8185		2015 n =6871		2016 n =7843		2017 n =7339	
	n	weighted % (95% CI)	n	weighted % (95% CI)	n	weighted % (95% CI)	n	weighted % (95% CI)	n	weighted % (95% CI)
**Hispanics**										
Never / non-current	1508	86.7 (84.0, 88.9)	1841	82.1 (77.6, 85.9)	1611	80.7 (77.8, 83.4)	2037	87.6 (84.5, 90.1)	1736	88.6 (83.5, 92.3)
Exclusive menthol cigarette use	66	4.0 (2.8, 5.5)	25	1.1 (0.7, 1.7)	38	2.0 (1.3, 3.0)	24	0.9 (0.5, 1.6)	25	1.2 (0.7, 2.1)
Exclusive non-menthol cigarette use	80	4.8 (3.7, 6.4)	42	1.9 (1.3, 2.7)	38	2.1 (1.4, 3.0)	35	1.6 (1.0, 2.6)	32	1.5 (0.9, 2.3)
Exclusive ENDS use	33	1.9 (1.3, 2.7)	225	9.8 (7.2, 13.1)	224	12.0 (9.8, 14.5)	140	6.7 (5.5, 8.2)	110	5.9 (3.9, 8.8)
Dual use of ENDS + menthol cigarettes	30	1.5 (0.9, 2.7)	57	3.0 (1.9, 4.6)	35	1.7 (1.1, 2.8)	34	1.8 (1.2, 2.8)	15	0.9 (0.4, 1.8)
Dual use of ENDS + non-menthol cigarettes	17	1.1 (0.7, 1.8)	51	2.2 (1.5, 3.3)	32	1.6 (1.1, 2.3)	28	1.4 (0.8, 2.5)	36	1.9 (1.2, 3.3)
**Non-Hispanic White**										
Never / non-current	2725	85.2 (83.0, 87.3)	2871	80.9 (77.6, 83.8)	2641	79.8 (76.8, 82.5)	2731	82.6 (80.4, 84.6)	2639	81.6 (79.0, 83.9)
Exclusive menthol cigarette use	160	4.3 (3.2, 5.6)	74	1.6 (1.0, 2.4)	44	1.1 (0.7, 1.8)	56	1.9 (1.2, 3.1)	67	1.8 (1.1, 2.9)
Exclusive non-menthol cigarette use	173	5.6 (4.6, 6.7)	104	2.6 (2.0, 3.3)	83	2.7 (2.0, 3.5)	80	2.8 (2.0, 3.8)	81	2.4 (1.8, 3.2)
Exclusive ENDS use	44	1.6 (1.1, 2.3)	313	8.7 (6.7, 11.3)	342	10.1 (8.6, 11.9)	265	8.1 (6.8, 9.4)	287	9.1 (7.4, 11.3)
Dual use of ENDS + menthol cigarettes	65	1.8 (1.1, 2.9)	136	3.4 (2.5, 4.5)	100	2.8 (2.1, 3.8)	72	2.0 (1.6, 2.5)	75	2.2 (1.6, 3.1)
Dual use of ENDS + non-menthol cigarettes	49	1.6 (1.2, 2.3)	109	2.9 (2.2, 3.9)	117	3.5 (2.5, 4.8)	79	2.6 (2.1, 3.4)	89	2.9 (2.0, 4.1)
**Non-Hispanic Black**										
Never / non-current	1160	92.2 (89.5, 94.3)	1173	91.5 (88.6, 93.7)	871	89.1 (86.1, 91.5)	1170	92.3 (90.3, 94.0)	1226	93.5 (91.4, 95.1)
Exclusive menthol cigarette use	24	2.0 (1.3, 3.2)	9	0.5 (0.3, 1.1)	8	0.7 (0.3, 1.9)	9	0.6 (0.3, 1.3)	7	0.8 (0.3, 1.8)
Exclusive non-menthol cigarette use	50	4.1 (3.0, 5.6)	31	2.0 (1.4, 2.9)	21	2.7 (1.0, 6.9)	20	1.2 (0.6, 2.1)	18	1.1 (0.6, 1.9)
Exclusive ENDS use	7	0.4 (0.2, 1.1)	55	4.3 (2.8, 6.8)	58	6.0 (4.4, 8.2)	51	4.5 (3.3, 6.0)	38	3.6 (2.4, 5.3)
Dual use of ENDS + menthol cigarettes	8	0.9 (0.4, 2.2)	9	0.6 (0.3, 1.3)	5	0.4 (0.2, 0.9)	10	0.8 (0.4, 1.7)	4	0.3 (0.1, 0.7)
Dual use of ENDS + non-menthol cigarettes	5	0.4 (0.1, 1.6)	17	1.0 (0.5, 1.8)	10	1.1 (0.6, 2.2)	8	0.7 (0.3, 1.8)	7	0.8 (0.4, 1.8)
**Non-Hispanic Other**										
Never / non-current	857	89.9 (86.6, 92.4)	880	83.5 (80.2, 86.2)	470	77.5 (68.7, 84.4)	902	90.3 (87.3, 92.7)	753	89.5 (84.9, 92.8)
Exclusive menthol cigarette use	31	2.5 (1.6, 3.8)	21	1.8 (1.0, 3.3)	12	2.1 (1.2, 3.7)	13	0.9 (0.4, 2.0)	15	1.7 (0.8, 3.6)
Exclusive non-menthol cigarette use	32	3.1 (1.9, 5.0)	24	1.9 (1.2, 3.0)	7	1.3 (0.4, 3.6)	11	1.1 (0.5, 2.7)	10	0.9 (0.4, 1.7)
Exclusive ENDS use	14	1.9 (0.8, 4.2)	82	8.9 (6.8, 11.6)	72	13.4 (8.6, 20.2)	40	4.7 (3.1, 6.9)	39	5.1 (3.1, 8.3)
Dual use of ENDS + menthol cigarettes	9	1.2 (0.5, 2.5)	19	2.1 (1.1, 3.9)	17	3.4 (1.8, 6.2)	11	1.4 (0.6, 3.2)	11	1.1 (0.5, 2.2)
Dual use of ENDS + non-menthol cigarettes	12	1.6 (0.6, 3.9)	17	1.8 (0.9, 3.3)	15	2.4 (1.4, 4.2)	17	1.6 (0.9, 2.7)	19	1.7 (0.9, 3.5)

Abbreviations: (95% CI) = 95% Confidence Interval

Table S7: Patterns of exclusive and dual use of ENDS and menthol/non-menthol cigarettes among 15-17-year-olds by parental education level in the Population Assessment of Tobacco and Health (PATH), 2013-2017

	Wave 1 (2013/14) n = 6539		Wave 2 (2014/15) n = 5521		Wave 3 (2015/16) n = 5674		Wave 4 (2016/17) n = 7333	
	n	weighted % (95% CI)	n	weighted % (95% CI)	n	weighted % (95% CI)	n	weighted % (95% CI)
**High School / GED / Less than High School**								
Never / non-current	2328	88.0 (86.7, 89.2)	1896	89.0 (87.4, 90.4)	1958	89.5 (88.1, 90.8)	2560	89.9 (88.6, 91.0)
Exclusive menthol cigarette use	111	4.2 (3.5, 5.1)	66	3.4 (2.6, 4.5)	51	2.3 (1.6, 3.2)	67	2.4 (1.9, 3.1)
Exclusive non-menthol cigarette use	72	2.9 (2.3, 3.6)	52	2.6 (1.8, 3.6)	35	1.9 (1.4, 2.7)	57	2.1 (1.6, 2.7)
Exclusive ENDS use	62	2.7 (2.0, 3.6)	55	2.6 (2.0, 3.5)	83	4.1 (3.2, 5.3)	94	3.9 (3.2, 4.8)
Dual use of ENDS + menthol cigarettes	45	1.7 (1.2, 2.3)	28	1.4 (1.0, 2.1)	26	1.3 (0.7, 2.2)	28	0.8 (0.5, 1.3)
Dual use of ENDS + non-menthol cigarettes	15	0.6 (0.4, 1.0)	20	1.0 (0.6, 1.5)	16	0.9 (0.5, 1.6)	25	0.9 (0.6, 1.5)
**Some College**								
Never / non-current	1901	89.4 (87.9, 90.8)	1593	88.7 (87.0, 90.3)	1625	88.2 (86.0, 90.0)	2075	89.2 (87.3, 90.7)
Exclusive menthol cigarette use	67	3.2 (2.5, 4.2)	43	2.4 (1.8, 3.3)	38	2.0 (1.5, 2.8)	39	1.7 (1.3, 2.4)
Exclusive non-menthol cigarette use	41	1.8 (1.3, 2.5)	53	2.7 (2.0, 3.6)	27	1.6 (1.1, 2.3)	45	1.8 (1.4, 2.5)
Exclusive ENDS use	51	2.6 (1.9, 3.4)	57	3.5 (2.7, 4.7)	86	5.2 (4.1, 6.7)	108	5.2 (4.2, 6.4)
Dual use of ENDS + menthol cigarettes	46	2.1 (1.4, 3.0)	18	1.0 (0.6, 1.7)	34	2.0 (1.3, 3.0)	29	1.2 (0.8, 1.8)
Dual use of ENDS + non-menthol cigarettes	20	0.9 (0.6, 1.5)	29	1.6 (1.0, 2.6)	19	1.0 (0.6, 1.8)	18	0.8 (0.5, 1.4)
**College or More**								
Never / non-current	1646	92.5 (91.2, 93.7)	1494	92.4 (91.1, 93.6)	1567	93.2 (91.8, 94.4)	1975	89.9 (88.3, 91.4)
Exclusive menthol cigarette use	32	1.7 (1.1, 2.5)	8	0.5 (0.2, 1.0)	8	0.5 (0.2, 1.1)	15	0.6 (0.4, 1.1)
Exclusive non-menthol cigarette use	25	1.5 (1.0, 2.4)	26	1.6 (1.0, 2.4)	21	1.3 (0.8, 2.0)	30	1.3 (0.9, 1.9)
Exclusive ENDS use	48	2.7 (2.0, 3.7)	54	3.8 (2.9, 4.9)	62	3.8 (2.8, 5.0)	131	6.0 (5.0, 7.2)
Dual use of ENDS + menthol cigarettes	14	0.7 (0.4, 1.3)	13	0.7 (0.4, 1.4)	10	0.7 (0.4, 1.3)	16	1.0 (0.6, 1.6)
Dual use of ENDS + non-menthol cigarettes	15	0.8 (0.5, 1.3)	16	1.0 (0.6, 1.6)	8	0.5 (0.3, 0.9)	21	1.1 (0.7, 1.9)

Abbreviations: (95% CI) = 95% Confidence Interval

Table S8: Patterns of exclusive and dual use of ENDS and menthol/non-menthol cigarettes among 15-17-year-olds by annual household income in the Population Assessment of Tobacco and Health (PATH), 2014-2017

	Wave 1 (2013/14)		Wave 2 (2014/15) n = 5372		Wave 3 (2015/16) n = 5542		Wave 4 (2016/17) n = 7187	
	n	weighted % (95% CI)	n	weighted % (95% CI)	n	weighted % (95% CI)	n	weighted % (95% CI)
**< $50,000**								
Never / non-current	NA		2413	89.0 (87.5, 90.3)	2442	88.7 (87.2, 90.1)	3126	89.6 (88.5, 90.7)
Exclusive menthol cigarette use	NA		82	3.2 (2.6, 4.0)	71	2.6 (2.0, 3.4)	89	2.6 (2.1, 3.2)
Exclusive non-menthol cigarette use	NA		78	3.0 (2.3, 3.8)	48	2.0 (1.5, 2.6)	76	2.2 (1.8, 2.8)
Exclusive ENDS use	NA		58	2.4 (1.8, 3.2)	105	4.2 (3.3, 5.3)	107	3.6 (2.9, 4.3)
Dual use of ENDS + menthol cigarettes	NA		33	1.3 (0.9, 1.9)	38	1.4 (1.0, 2.1)	40	1.1 (0.8, 1.5)
Dual use of ENDS + non-menthol cigarettes	NA		31	1.2 (0.8, 1.7)	25	1.1 (0.7, 1.7)	27	0.9 (0.6, 1.4)
**≥ $50,000**								
Never / non-current	NA		2439	91.0 (89.7, 92.2)	2583	91.4 (90.1, 92.6)	3344	89.5 (88.1, 90.7)
Exclusive menthol cigarette use	NA		31	1.2 (0.9, 1.7)	24	0.8 (0.5, 1.2)	31	0.8 (0.6, 1.2)
Exclusive non-menthol cigarette use	NA		50	1.7 (1.2, 2.3)	35	1.4 (1.0, 1.9)	54	1.4 (1.0, 1.9)
Exclusive ENDS use	NA		99	3.9 (3.1, 4.9)	121	4.5 (3.6, 5.6)	225	6.3 (5.5, 7.3)
Dual use of ENDS + menthol cigarettes	NA		24	0.9 (0.5, 1.4)	32	1.2 (0.8, 1.9)	33	1.0 (0.7, 1.4)
Dual use of ENDS + non-menthol cigarettes	NA		34	1.3 (0.9, 1.8)	18	0.7 (0.4, 1.1)	35	1.0 (0.7, 1.4)

Abbreviations: (95% CI) = 95% Confidence Interval

Question about annual household income was not asked in PATH Wave 1 (2013/14).

Table S9: Patterns of exclusive and dual use of ENDS and menthol/non-menthol cigarettes among 15-17-year-olds by homeownership status in the Population Assessment of Tobacco and Health (PATH), 2014-2017

	Wave 1 (2013/14)		Wave 2 (2014/15) n = 5510		Wave 3 (2015/16) n = 5675		Wave 4 (2016/17) n = 7335	
	n	weighted % (95% CI)	n	weighted % (95% CI)	n	weighted % (95% CI)	n	weighted % (95% CI)
**Home Not Owned**								
Never / non-current		NA	1947	88.7 (87.2, 90.0)	2040	88.9 (87.2, 90.3)	2653	89.7 (88.4, 90.8)
Exclusive menthol cigarette use		NA	70	3.4 (2.7, 4.2)	60	2.6 (1.9, 3.5)	78	2.7 (2.1, 3.4)
Exclusive non-menthol cigarette use		NA	60	2.7 (2.0, 3.7)	37	1.8 (1.3, 2.6)	65	2.2 (1.7, 2.9)
Exclusive ENDS use		NA	56	2.7 (2.1, 3.6)	88	4.2 (3.3, 5.3)	89	3.8 (3.0, 4.7)
Dual use of ENDS + menthol cigarettes		NA	26	1.2 (0.8, 1.7)	35	1.5 (0.9, 2.3)	34	1.1 (0.7, 1.6)
Dual use of ENDS + non-menthol cigarettes		NA	29	1.4 (0.9, 2.1)	21	1.0 (0.6, 1.7)	19	0.7 (0.4, 1.1)
**Home Owned**								
Never / non-current		NA	3025	90.8 (89.5, 91.9)	3111	91.2 (90.0, 92.3)	3958	89.6 (88.5, 90.7)
Exclusive menthol cigarette use		NA	47	1.4 (1.1, 1.9)	37	1.0 (0.7, 1.4)	43	1.0 (0.7, 1.3)
Exclusive non-menthol cigarette use		NA	71	2.0 (1.5, 2.6)	46	1.5 (1.1, 1.9)	67	1.5 (1.2, 1.9)
Exclusive ENDS use		NA	110	3.7 (3.0, 4.5)	143	4.5 (3.6, 5.5)	245	5.8 (5.0, 6.7)
Dual use of ENDS + menthol cigarettes		NA	33	1.0 (0.7, 1.5)	35	1.2 (0.8, 1.8)	39	0.9 (0.7, 1.3)
Dual use of ENDS + non-menthol cigarettes		NA	36	1.1 (0.7, 1.6)	22	0.7 (0.5, 1.0)	45	1.2 (0.8, 1.6)

Abbreviations: (95% CI) = 95% Confidence Interval
Question about homeownership was not asked in PATH Wave 1 (2013/14).

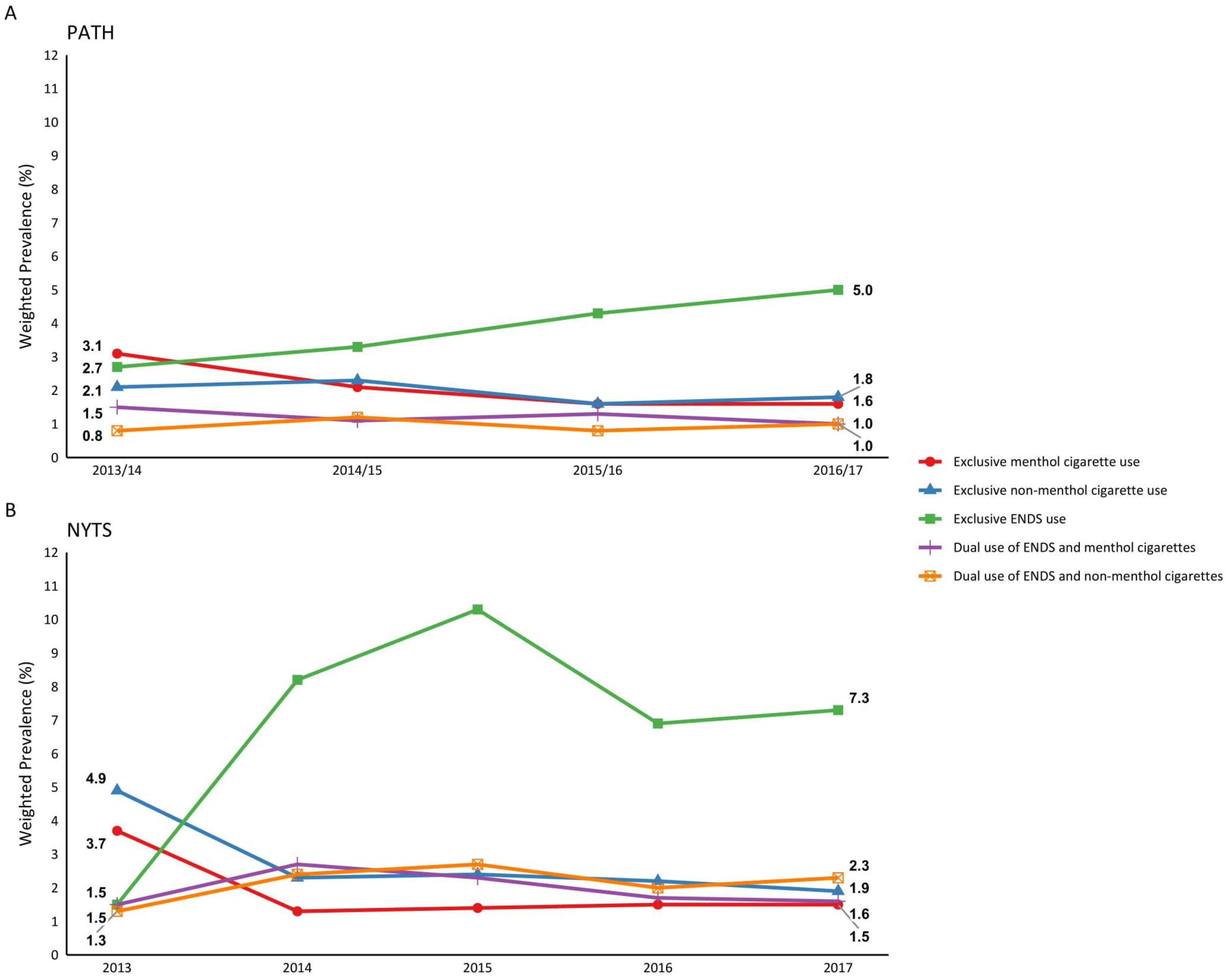

Figure S1. Prevalence of exclusive and dual use of ENDS and menthol/non-menthol cigarettes overall in (A) PATH and (B) NYTS.

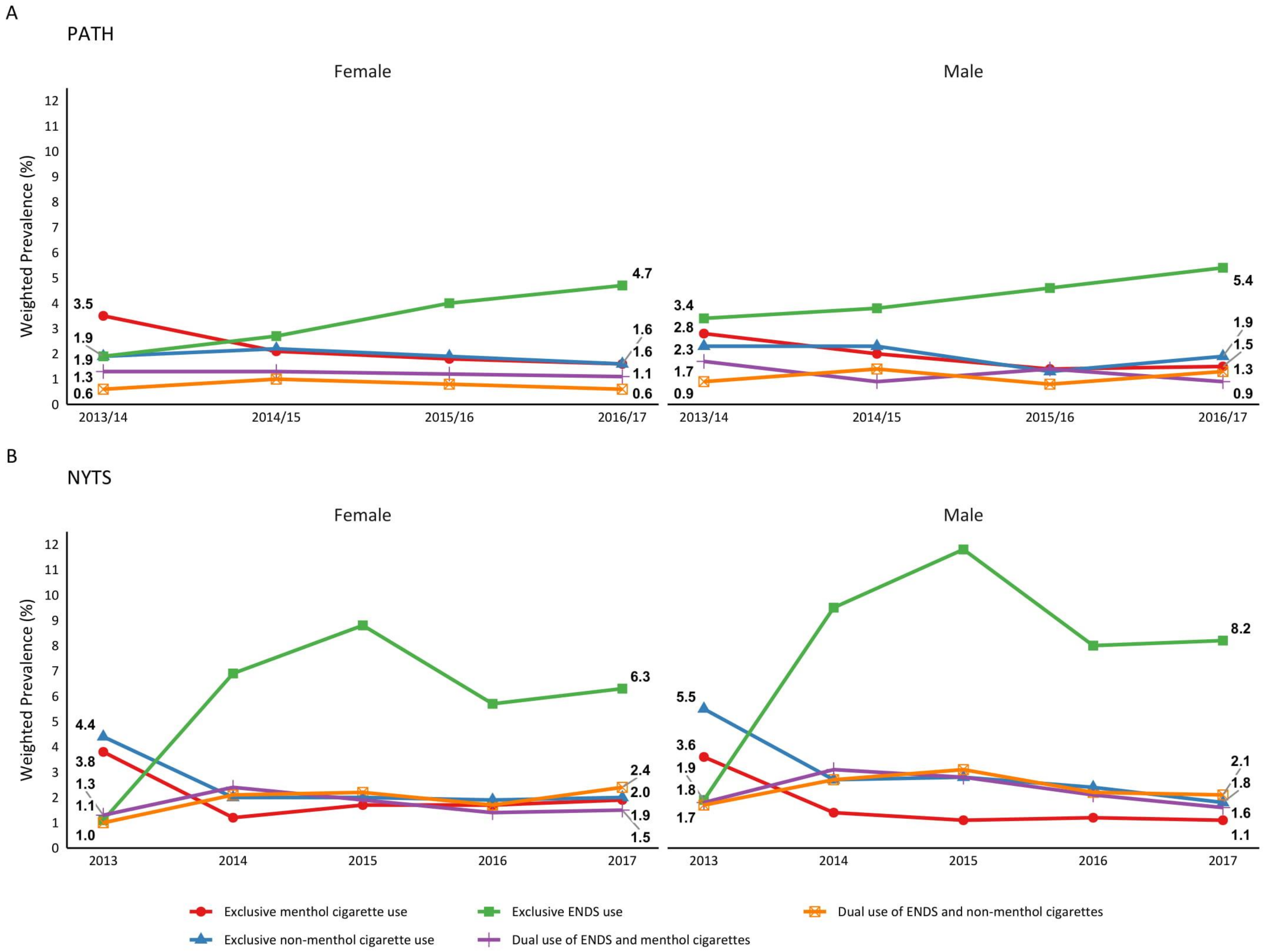

Figure S2. Prevalence of exclusive and dual use of ENDS and menthol/non-menthol cigarettes by sex in (A) PATH and (B) NYTS.

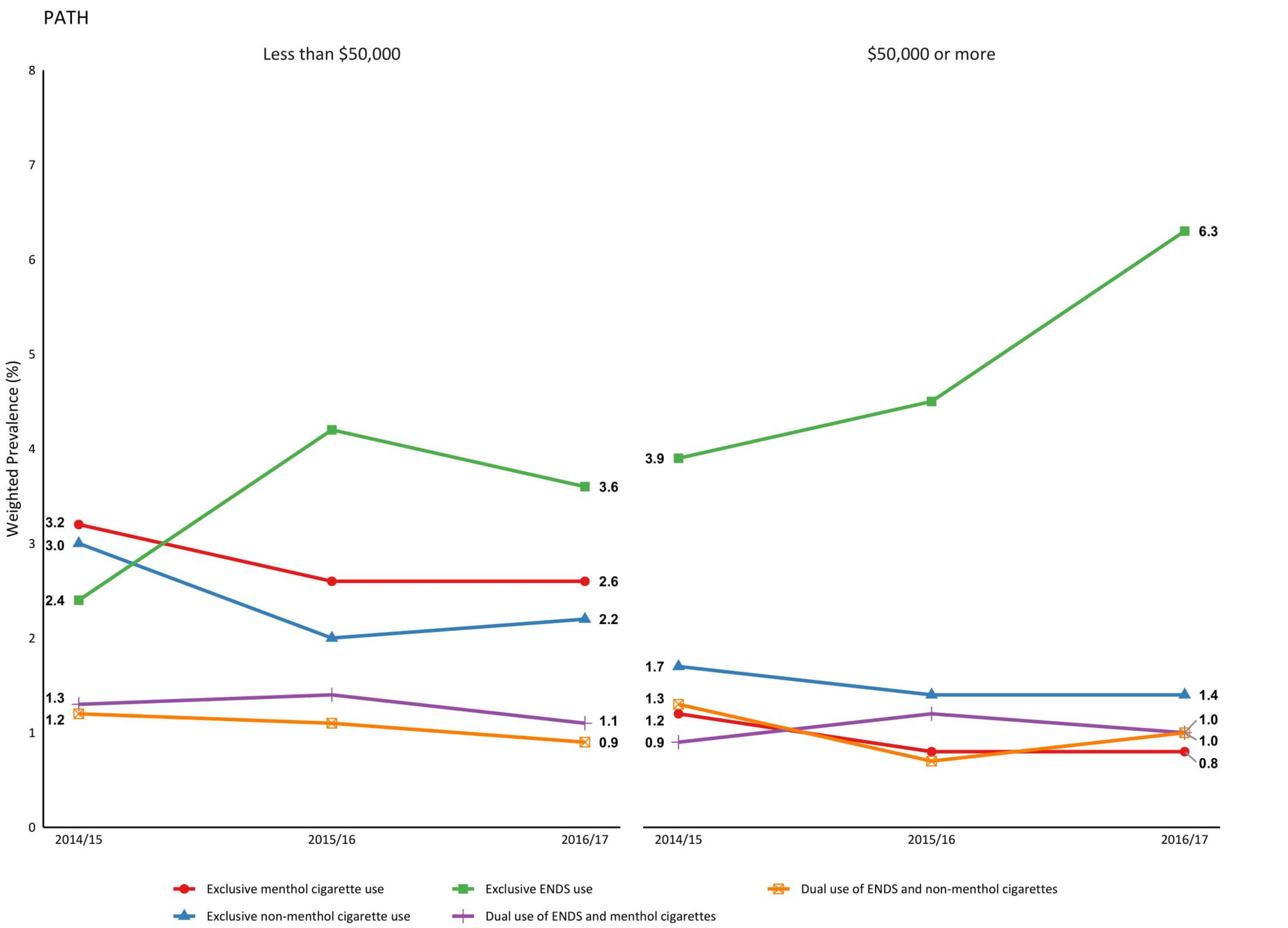

Figure S3. Prevalence of exclusive and dual use of ENDS and menthol/non-menthol cigarettes overall by household income in PATH. Information about household income was not collected in PATH Wave 1 (2013/14).

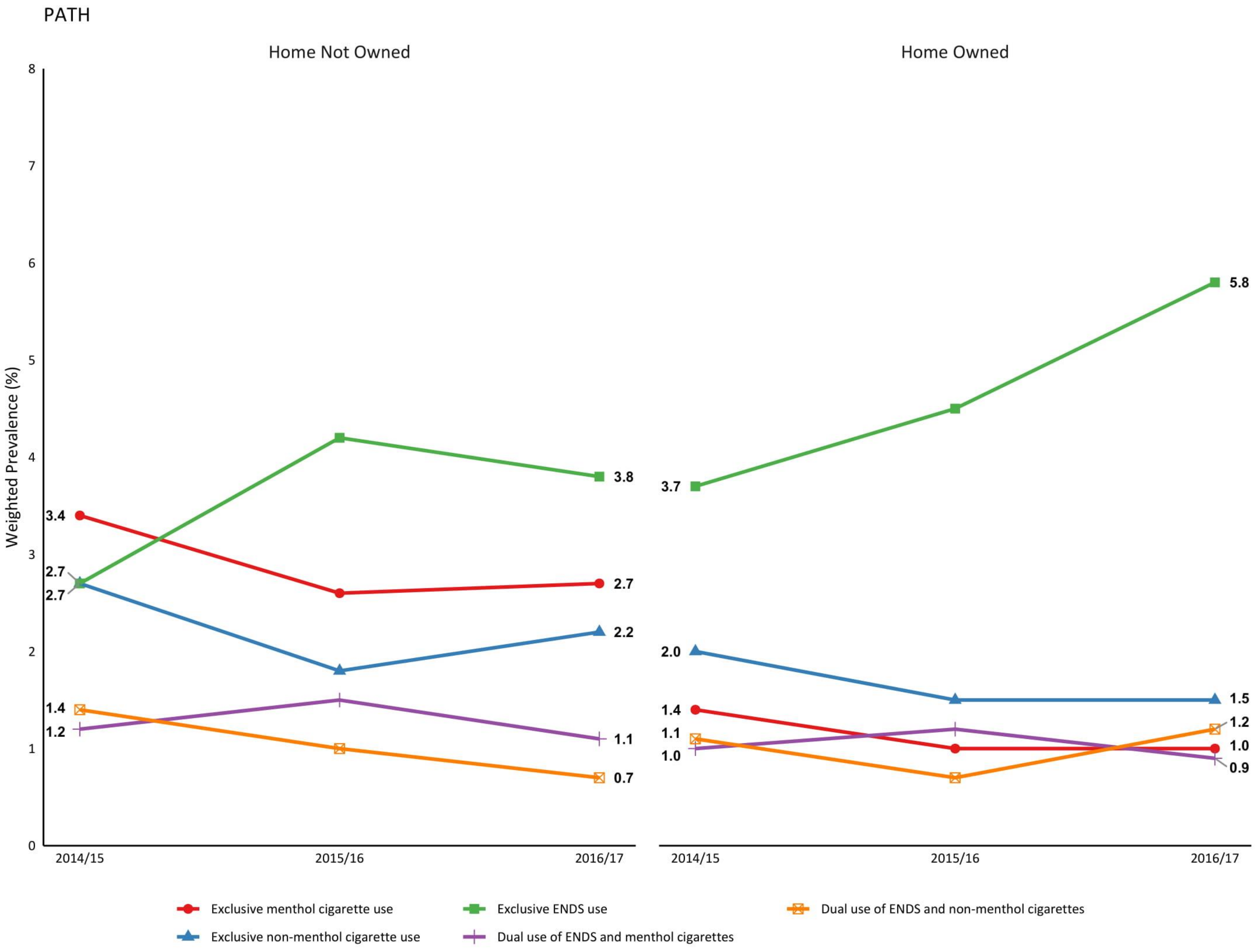

Figure S4. Prevalence of exclusive and dual use of ENDS and menthol/non-menthol cigarettes overall by homeownership in PATH. Information about homeownership was not collected in PATH Wave 1 (2013/14).
